# Data on plasma tumour and metabolism related proteins’ potential in differentiation of HFpEF-PH from PAH and in prognosis of left heart failure patients with pulmonary hypertension

**DOI:** 10.1016/j.dib.2021.107747

**Published:** 2021-12-22

**Authors:** Salaheldin Ahmed, Abdulla Ahmed, Göran Rådegran

**Affiliations:** a Department of Clinical Sciences Lund, Cardiology, Lund University, Lund, Sweden; b The Haemodynamic Lab, The Section for Heart Failure and Valvular Disease, Heart and Lung Medicine, Skåne University Hospital, Lund, Sweden

**Keywords:** Prognosis, HFpEF, Pulmonary hypertension, Pulmonary hypertension due to left heart disease, PAH, Haemodynamics, Proteomics, Cancer

## Abstract

The data in the current paper constitutes supplementary material to our article entitled “Plasma tumour and metabolism related biomarkers AMBP, LPL and Glyoxalase I differentiate heart failure with preserved ejection fraction with pulmonary hypertension from pulmonary arterial hypertension” Ahmed et al. (2021). The study investigated 69 plasma tumour- and metabolism related proteins in healthy controls (*n* = 20) and in 115 patients of whom 48 had pulmonary arterial hypertension (PAH; *n* = 48) and 67 with left heart failure with pulmonary hypertension (LHF-PH) [heart failure with- preserved ejection fraction-PH (HFpEF-PH; *n* = 31) and reduced ejection fraction-PH (HFrEF-PH; *n* = 36)]. The haemodynamic data were obtained with right heart catheterization, and clinical data from medical records. The present article describe the plasma levels of tumour- and metabolism related proteins, analyzed with proximity extension assay, along with their uni- and multivariable diagnostic and prognostic potential. High sRAGE levels univariably emerged as a negative prognostic marker in LHF-PH.

## Specifications Table

**Table:** 

Subject	Cardiology and Cardiovascular Medicine.
Specific subject area	Pulmonary hypertension and heart failure with preserved ejection fraction.
Type of data	Text, tables, and figures.
How the data were acquired	Hemodynamic data were acquired with right heart catheterisation. Clinical data were obtained from clinical examinations and medical records. Venous blood samples were collected upon enrolment and/or during the right heart catheterization procedures. Proximity extension assay was used to quantify the proteins’ levels. Statistical tests were performed with R version 4.0.2, (Foundation for Statistical Computing, Vienna, Austria) and GraphPad Prism version 8.4.1 for Windows, (GraphPad Software, San Diego, California USA.
Data format	Raw, analysed and filtered.
Description of data collection	Clinical data and blood samples were collected prospectively from healthy controls, as well as from patients with pulmonary arterial hypertension and left heart failure with pulmonary hypertension during the routine clinical evaluations involving haemodynamic assessments. All participants were >18 years of age. Clinical data were collected from medical records.
Data source location	Skåne University Hospital, Lund, Sweden.
Data accessibility	Data access can be requested by e-mail to corresponding author. For ethical reasons, a user-specific data use agreement can be set up upon request when a valid scientific purpose is provided along with a clear clinical benefit.
Related research article	Ahmed S, Ahmed A, Rådegran G (2021) Plasma tumour and metabolism related biomarkers AMBP, LPL and Glyoxalase I differentiate heart failure with preserved ejection fraction with pulmonary hypertension from pulmonary arterial hypertension. Int J Cardiol 345:68–76 [1]. https://doi.org/10.1016/j.ijcard.2021.10.136

## Value of the Data

• The data outline the potential of tumour- and metabolism related proteins in the diagnostic differentiation of heart failure with preserved ejection fraction with pulmonary hypertension (HFpEF-PH) from pulmonary arterial hypertension (PAH). The data also describe the potential of such proteins in predicting transplantation-free survival in patients with left heart failure and pulmonary hypertension (LHF-PH).
• These data may aid clinical professionals working in the field of pulmonary hypertension in decision making to avoid or initiate PAH-specific therapy early to improve survival and quality of life in patients with PAH.
• The present data provide cardiologists/pulmonologists and researchers working in the field of pulmonary hypertension and/or heart failure a basis for further investigations aimed at facilitating the diagnostic differentiation between PAH and HFpEF-PH.
• Using these data, clinicians and researchers can obtain further insights into the potential utility of proteomics in diagnosis and prognosis of LHF-PH and PAH.

## 1 Data Description

### 1.1 Population characteristics

**Fig. 1:**
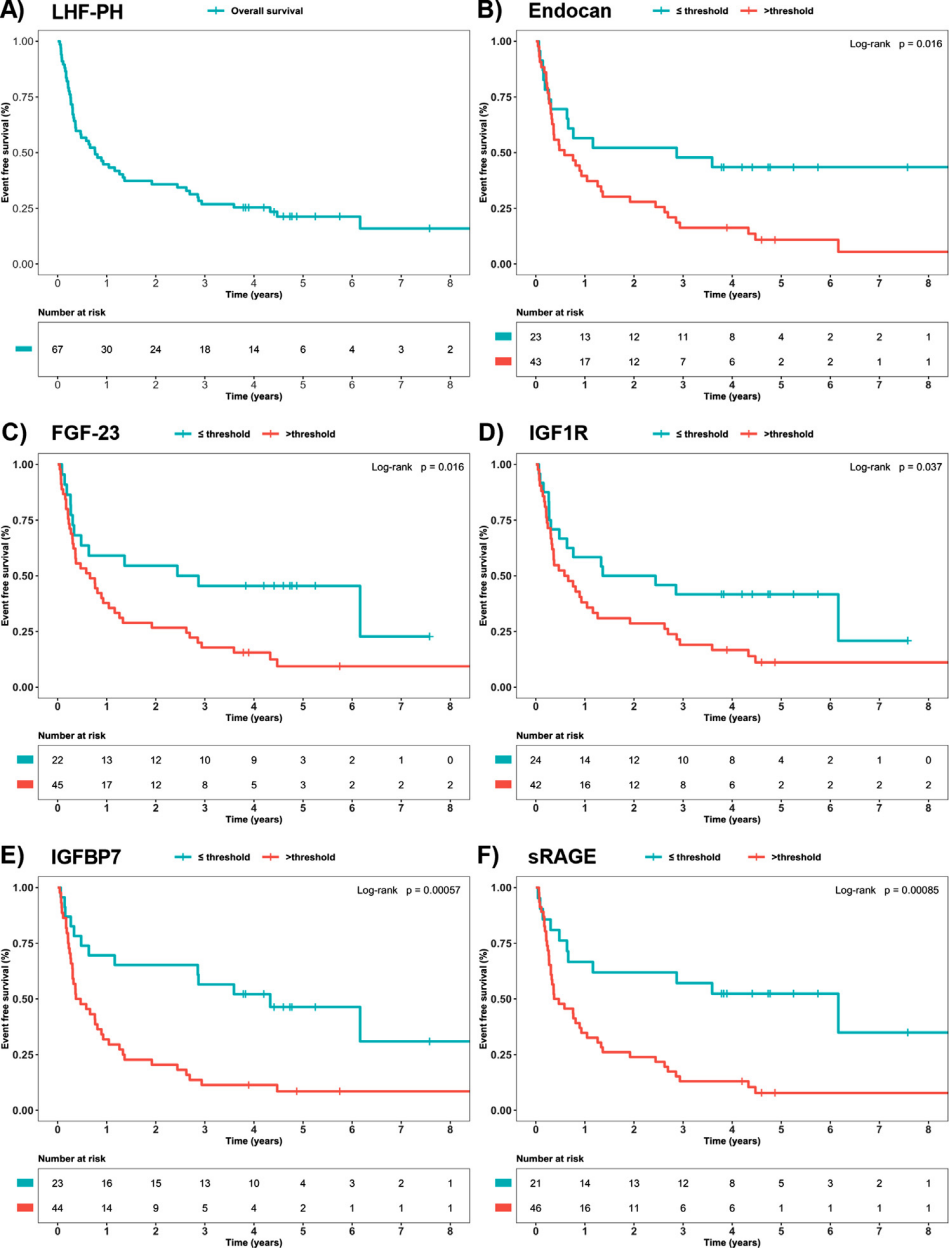
Kaplan-Meier analysis of transplantation-free survival in patients with LHF-PH. **(A)** Overall survival of patients with left heart failure with pulmonary hypertension (LHF-PH). **(B–F)** Survival based on dichotomized plasma levels of endocan, fibroblast growth factor 23 (FGF-23), insulin-like growth factor 1 receptor (IGF1R), insulin-like growth factor-binding protein 7 (IGFBP7) and soluble receptor for advanced glycation end products (sRAGE) with log-rank p-values illustrated at the top left, respectively. Censoring (defined as the end-of-study follow-up time) is illustrated as upright slashes and the numbers at risk are presented on a table below each plot, respectively. The protein specific thresholds were identified with receiver operating characteristic analyses of event-free survivors vs non-dittos in LHF-PH.

During the total study follow-up between 31 October 2011 and 21 August 2020, [n (%)] 25 (37.3%) patients with left heart failure with pulmonary hypertension (LHF-PH) died, of which 16 (51.6%) had heart failure with preserved ejection fraction with pulmonary hypertension (HFpEF-PH) and 9 (29%) heart failure with reduced ejection fraction with pulmonary hypertension (HFrEF-PH). Thirty-six (53.7%) patients with LHF-PH were heart transplanted, of which 1 (3.2) had HFpEF-PH and 35 (92.7%) HFrEF-PH. A total of 53 (79.1) events occurred in LHF-PH, of which 17 (54.8) had HFpEF-PH and 36 (100%) HFrEF-PH. The median transplantation-free survival was 279 (96–1382), 1382 (486–1743) and 120 (70.3–268) days for LHF-PH, HFpEF-PH and HFrEF-PH, respectively (Fig. 1A). A more thorough description of the study population and a more detailed overview of the study setup can be found in the associated publication in *Table 1* and *Fig. 1,* respectively [1].

### 1.2 The plasma levels of tumour- and metabolism related proteins

The plasma levels ‒ expressed in linear normalized protein expression (NPX) scale as arbitrary units (AU) ‒ in the study group, as well as heart failure specific classification of tumour- and metabolism related proteins are described in (Tables 1 and 2).

**Table 1:** Plasma levels of metabolism and tumour related proteins in controls and patients.

Protein (AU)	Control (*n* = 20)	LHF-PH (*n* = 67)	HFpEF-PH (*n* = 31)	HFrEF-PH (*n* = 36)	PAH (*n* = 48)
	Median (IQR)	Median (IQR)	Median (IQR)	Median (IQR)	Median (IQR)
5′-NT	655.4 (535.21 ‒ 716.19)#	1515.4 (889.94 ‒ 2172.2)#	1018.2 (685.17 ‒ 2079.7)	1636 (1084.9 ‒ 2234.6)#	1107.5 (748.56 ‒ 1778.5)
AMBP	96.07 (87.51 ‒ 108.12)	111.84 (98.19 ‒ 125.42)	121.72 (103.9 ‒ 131.65)	105.29 (94.39 ‒ 117.04)	100.52 (91.82 ‒ 112.02)
AP-N	20.98 (18.93 ‒ 23.63)	24.47 (19.7 ‒ 32.71)	23.33 (18.41 ‒ 31.17)	25.51 (21.28 ‒ 34.74)	20.05 (16.56 ‒ 28.68)
BLM-H	31.82 (26.24 ‒ 37.1)	32.97 (28.03 ‒ 39.09)	31.17 (27.54 ‒ 39.7)	33.57 (28.2 ‒ 38.93)	29.3 (22.99 ‒ 36.47)
BOC	27.68 (24.44 ‒ 32.58)	31.26 (26.64 ‒ 37.7)	28.1 (23.98 ‒ 32.97)	34.08 (28.98 ‒ 45.61)	24.31 (20.62 ‒ 31)
CA9	5.08 (3.37 ‒ 6.16)#	10.57 (6.2 ‒ 17.67)#	10.73 (6.25 ‒ 19.29)	10.41 (4.78 ‒ 15.83)#	9.29 (7.1 ‒ 15.09)
Cathepsin Z	18.81 (15.72 ‒ 21.55)	21.32 (17.62 ‒ 27.56)	24.12 (18.08 ‒ 31.73)	20.14 (16 ‒ 23.59)	19.27 (14.99 ‒ 23.04)
CDKN1A	24.69 (9.24 ‒ 36.37)#	17.33 (11.3 ‒ 33.17)#	24.62 (14.32 ‒ 53.63)	13.41 (7.26 ‒ 29.8)#	11.93 (7.89 ‒ 33.78)
CEACAM1	132.01 (123.7 ‒ 138.46)#	144.66 (130.8 ‒ 165.77)#	134.55 (129.23 ‒ 156.76)	154.11 (137.43 ‒ 168.94)#	129.01 (118.01 ‒ 140.79)
CEACAM5	3.38 (2.54 ‒ 6.9)#	4.33 (3.16 ‒ 6.71)#	5.28 (3.33 ‒ 10.56)	3.68 (2.72 ‒ 5.35)#	4.88 (3.54 ‒ 7.01)
Contactin-1	6.04 (4.98 ‒ 6.68)	4.85 (4.26 ‒ 5.84)	5.45 (4.3 ‒ 6.36)	4.78 (4.23 ‒ 5.69)	4.78 (3.79 ‒ 5.75)
Cornulin	36.69 (28.77 ‒ 56.91)#	22.57 (12.27 ‒ 32.47)#	26.96 (15.87 ‒ 39.06)	16.08 (10.17 ‒ 25.28)#	22.45 (15.26 ‒ 30.72)
CPA1	15.12 (10.45 ‒ 21.53)	25.39 (16 ‒ 35.58)	20.9 (13.51 ‒ 35.58)	27.71 (17.69 ‒ 36.62)	17.41 (11.88 ‒ 23.42)
CPB1	11.65 (7.69 ‒ 14.77)	17.85 (11.14 ‒ 22.75)	14.98 (8.17 ‒ 20.87)	19.8 (12.73 ‒ 28.49)	13.77 (7.93 ‒ 18.5)
CPE	9.77 (7.19 ‒ 11.41)#	11.4 (8.99 ‒ 14.03)#	9.49 (8.42 ‒ 11.08)	12.76 (11.27 ‒ 14.71)#	9.73 (8.78 ‒ 11.13)
Cystatin B	13.77 (10.18 ‒ 20.12)	30.93 (21.44 ‒ 43.18)	31.25 (26.62 ‒ 40.28)	30.01 (20.18 ‒ 45.12)	25.3 (16.87 ‒ 36.93)
Decorin	94.86 (39.73 ‒ 200.48)	64.73 (29.83 ‒ 115.58)	83.89 (42.95 ‒ 133.03)	47.18 (22.96 ‒ 106.03)	71.36 (38.57 ‒ 145.46)
Endocan	320.07 (261.24 ‒ 387.79)#	542.4 (440.2 ‒ 701.54)#	476.63 (395.19 ‒ 625.64)	616.32 (462.56 ‒ 789.1)#	423.04 (342.87 ‒ 511.15)
EpCAM	9.06 (7.57 ‒ 28.8)	11.56 (6.56 ‒ 22.01)	13.15 (8.62 ‒ 21.08)	10.31 (6.33 ‒ 24.48)	8.33 (5.52 ‒ 13.8)
FABP4	9.83 (7.93 ‒ 20.42)	57.85 (32.79 ‒ 96.28)	57.85 (31.98 ‒ 98.89)	60.09 (33.5 ‒ 95.07)	35.07 (26.78 ‒ 58.71)
FGF-21	34.46 (12.31 ‒ 65.53)	283.49 (100.16 ‒ 461.64)	253 (67.31 ‒ 387.54)	288.62 (179.27 ‒ 668.09)	195.26 (90.7 ‒ 368.96)
FGF-23	13.92 (11.05 ‒ 16.06)	74.27 (35.35 ‒ 289.85)	46.58 (21.59 ‒ 103)	110.09 (44.76 ‒ 566.45)	45.02 (27.52 ‒ 79.24)
FR-gamma	78.88 (73.41 ‒ 88.88)#	96.07 (82.31 ‒ 119.11)#	96.17 (83.95 ‒ 116.6)	90.43 (80.38 ‒ 2995.3)#	86.36 (76.36 ‒ 107.47)
Furin	8.45 (7.36 ‒ 11.08)#	11.29 (9.31 ‒ 13.59)#	11.51 (9.49 ‒ 14.8)	10.95 (9.2 ‒ 12.59)#	10.38 (9.02 ‒ 12.34)
Gastrotropin	2.23 (1.86 ‒ 2.8)	3.55 (2.75 ‒ 5.63)	3.36 (2.59 ‒ 5.63)	3.63 (2.81 ‒ 5.94)	2.65 (2.03 ‒ 3.62)
Glyoxalase I	83.54 (54.95 ‒ 178.74)	237.44 (166.12 ‒ 319.51)	238.38 (166.12 ‒ 316.12)	220.03 (162.71 ‒ 327.34)	85.79 (56.93 ‒ 256.34)
IGF1R	8.57 (7.86 ‒ 9.8)#	12.75 (10.84 ‒ 17.65)#	11.99 (10.79 ‒ 14.04)	13.88 (11.26 ‒ 18.15)#	10.38 (8.82 ‒ 12.08)
IGFBP1	8.23 (4.21 ‒ 24.14)	36.38 (21.06 ‒ 65.24)	36.15 (15.02 ‒ 59.2)	38.48 (21.43 ‒ 67.15)	26.1 (17.8 ‒ 56.87)
IGFBP2	131.47 (91.55 ‒ 183.84)	262.94 (185.3 ‒ 390.54)	262.94 (205.04 ‒ 377.13)	262.01 (170.17 ‒ 397.41)	257.49 (185.96 ‒ 323.95)
IGFBP7	12.35 (10.02 ‒ 13.58)	23.17 (17.4 ‒ 38.9)	18.42 (15.69 ‒ 29.02)	28.31 (19.64 ‒ 45.1)	15.4 (12.51 ‒ 21.13)
Kallikrein 11	27.45 (22.41 ‒ 33.3)#	55.1 (41.55 ‒ 65.17#	50.94 (35.54 ‒ 59.66)	55.61 (46.81 ‒ 75.28)#	36.83 (30.15 ‒ 48.05)
Kallikrein 13	22.49 (17.11 ‒ 30.63)#	32.21 (24.64 ‒ 42.67)#	29.79 (24.28 ‒ 45.78)	33.33 (24.78 ‒ 42.1)#	25.69 (19.14 ‒ 38.17)
Kallikrein 14	71 (52.03 ‒ 79.5)#	76.86 (61.47 ‒ 97.1)#	72.24 (57.57 ‒ 100.19)	79.19 (65.5 ‒ 96.39)#	79.8 (64.05 ‒ 105.82)
Kallikrein 6	6.46 (5.8 ‒ 7.66)	8.35 (7.42 ‒ 10.29)	8.12 (6.62 ‒ 9.83)	8.93 (7.85 ‒ 11.15)	7.37 (6.38 ‒ 8.69)
Kallikrein 8	52.96 (41.72 ‒ 62.14)#	57.61 (45.09 ‒ 73.03)#	54.35 (43.13 ‒ 72.94)	60.35 (49.57 ‒ 73.31)#	44.9 (37.61 ‒ 53.27)
LDL-R	12.22 (8.06 ‒ 15)	9.25 (6.82 ‒ 13.93)	8.68 (6.82 ‒ 14.16)	9.88 (6.86 ‒ 13.76)	9.74 (7.24 ‒ 14.47)
Leptin	31.29 (15.45 ‒ 84.37)	77.45 (35.38 ‒ 149.19)	100.43 (51.17 ‒ 201.54)	71.41 (21.48 ‒ 128.92)	81.89 (45.87 ‒ 111.55)
LOX-1	62.65 (52.31 ‒ 79.56)	104.24 (80.39 ‒ 135.79)	90.62 (74.24 ‒ 129.55)	106.45 (86.75 ‒ 137.37)	113.62 (83.89 ‒ 164.17)
LPL	987.39 (924.84 ‒ 1142.6)	1140 (850.61 ‒ 1378.6)	1216.1 (1132.2 ‒ 1465.3)	965.77 (757.99 ‒ 1297.1)	908.13 (752.15 ‒ 1087.4)
LYPD3	14.09 (11.29 ‒ 18.41)#	10.9 (9.62 ‒ 13.06)#	10.66 (9.74 ‒ 13.33)	11.25 (8.77 ‒ 12.78)#	12.16 (9.56 ‒ 15.19)
Mesothelin	2.87 (2.23 ‒ 4.7)#	4.48 (3.54 ‒ 7.38)#	4.33 (2.84 ‒ 7.41)	4.73 (3.66 ‒ 7.1)#	5.53 (3.59 ‒ 8.38)
MetAP2	31.34 (16.74 ‒ 37.82)#	24.25 (18.59 ‒ 37.69)#	26.56 (20.32 ‒ 38.54)	23 (17.85 ‒ 36.21)#	23.12 (17.12 ‒ 34.68)
MIA	796.08 (722.87 ‒ 895.82)#	824.01 (727.68 ‒ 900.93)#	801.25 (692.86 ‒ 862.38)	845.87 (750.84 ‒ 938.4)#	825.86 (763.18 ‒ 892.3)
Midkine	72.06 (50.74 ‒ 84.89)#	115 (85.35 ‒ 152.78)#	121.46 (98.75 ‒ 147.08)	110.22 (76.82 ‒ 170.53)#	106.14 (79.26 ‒ 139.59)
Mucin 16	14.52 (11.6 ‒ 20.74)#	24.66 (14.81 ‒ 123.6)#	19.3 (11.68 ‒ 43.33)	66.46 (19.01 ‒ 214.16)#	14.63 (9.4 ‒ 26.3)
Nectin-4	35.51 (33.09 ‒ 40.83)#	51.19 (40.38 ‒ 66.02)#	52.27 (42.61 ‒ 73.44)	49.08 (35.75 ‒ 62.69)#	49.11 (36.01 ‒ 56.9)
Pappalysin-1	9.27 (7.76 ‒ 10.72)	14.27 (11.06 ‒ 17.63)	13.62 (11.25 ‒ 17.44)	14.52 (10.66 ‒ 19.91)	10.59 (7.74 ‒ 14.77)
PCSK9	3.07 (2.88 ‒ 3.68)	3.63 (3.21 ‒ 4.32)	3.63 (3.22 ‒ 4.34)	3.63 (3.16 ‒ 4.3)	3.6 (2.99 ‒ 4.03)
Podocalyxin	9.6 (9.22 ‒ 10.89)#	9.23 (8.25 ‒ 10.45)#	9.29 (8.57 ‒ 10.97)	9.2 (8.02 ‒ 10.27)#	9.44 (8.28 ‒ 10.62)
PON3	57.03 (43.46 ‒ 104.41)	24.81 (17.62 ‒ 33.01)	24.68 (17.29 ‒ 37.19)	24.85 (17.64 ‒ 32.74)	23.62 (17.21 ‒ 37.48)
Prostasin	289.25 (238.64 ‒ 337.37)	456.76 (375.52 ‒ 516.8)	469.07 (382.79 ‒ 529.95)	449.89 (374.22 ‒ 501.68)	436.22 (332.32 ‒ 522.22)
RARRES2	2163.6 (1901.1 ‒ 2241.2)	2657.7 (2378 ‒ 2937.2)	2789 (2408.4 ‒ 3142.9)	2541.7 (2329.5 ‒ 2821.1)	2568.1 (2029.5 ‒ 2921.1)
Resisin	53.17 (42.74 ‒ 65.61)	73 (53.51 ‒ 97.61)	82.68 (63.76 ‒ 97.61)	60.83 (50.02 ‒ 97.96)	68.32 (51.4 ‒ 84.18)
S100A11	4.77 (4.52 ‒ 5.17)#	6.14 (5.45 ‒ 7.24)#	6.27 (5.61 ‒ 7.62)	5.81 (5.42 ‒ 7.09)#	6.2 (5.29 ‒ 7.1)
S100A4	3.72 (2.81 ‒ 4.37)#	3.32 (2.83 ‒ 4.16)#	3.58 (3.19 ‒ 4.41)	3.04 (2.65 ‒ 3.57)#	3.26 (2.94 ‒ 3.91)
SCAMP3	46.14 (12.63 ‒ 60.66)#	31.89 (16.74 ‒ 54.79)#	37.73 (25.09 ‒ 73.26)	23.5 (13.07 ‒ 47.11)#	26.21 (14.08 ‒ 61.52)
SCGB3A2	3.64 (2.92 ‒ 4.38)	6.7 (4.32 ‒ 8.99)	7.6 (4.71 ‒ 9.23)	5.47 (4.27 ‒ 8.21)	6.57 (3.25 ‒ 10.64)
SERPIN A12	6.91 (3.68 ‒ 11.87)	10.86 (6.89 ‒ 18.26)	9.34 (4.98 ‒ 13.8)	13.7 (6.97 ‒ 21.32)	7.42 (5.49 ‒ 11.94)
SHPS-1	7.85 (7.06 ‒ 10.04)	11.16 (8.05 ‒ 14.32)	11.16 (7.98 ‒ 14.32)	11.04 (8.05 ‒ 14.43)	8.87 (6.75 ‒ 10.63)
Sortillin	53.7 (48.13 ‒ 63.56)	67.49 (61.79 ‒ 74.7)	68.09 (62.41 ‒ 75.48)	67.27 (61.26 ‒ 74.11)	62.32 (56.69 ‒ 74.18)
sRAGE	27.05 (25.08 ‒ 29.76)	52.93 (42.12 ‒ 66.26)	44.4 (36.37 ‒ 52.93)	62.38 (50.89 ‒ 70.01)	38.93 (29.72 ‒ 46.74)
TCL1A	53.01 (30.51 ‒ 91.59)#	28.1 (13.63 ‒ 57.41)#	30.75 (16.97 ‒ 64.45)	27.05 (13.01 ‒ 57.14)#	47.26 (24.81 ‒ 78.37)
TFF3	20.76 (18.69 ‒ 25.81)	44.66 (35 ‒ 63.96)	44.66 (35.94 ‒ 63.96)	43.96 (33.84 ‒ 65.22)	45.09 (34 ‒ 55.94)
TGM2	247.2 (133.97 ‒ 325.07)	184.93 (144.54 ‒ 230.34)	184.93 (139.64 ‒ 210.12)	187.76 (150.65 ‒ 240.78)	260.46 (199.55 ‒ 369.44)
TR	14.9 (12.52 ‒ 17)	20.26 (14.36 ‒ 27.7)	20.26 (14.36 ‒ 24.19)	19.67 (14.06 ‒ 31.62)	18.03 (12.23 ‒ 32.69)
WFDC2	68.77 (59 ‒ 76.82)#	167.81 (136.84 ‒ 210.06)#	157.8 (138.87 ‒ 205.11)	176.87 (134.89 ‒ 215.96)#	179.8 (138.82 ‒ 206.61)
Vimentin	6.02 (3.96 ‒ 9.44)#	15.56 (12.01 ‒ 20.83)#	13.78 (11.82 ‒ 19.7)	16.62 (12.77 ‒ 22.49)#	15.48 (11.2 ‒ 19.75)
VSIG2	7.33 (6.84 ‒ 8.41)	16.53 (12.36 ‒ 23.16)	17.8 (12.42 ‒ 25.16)	15.64 (12.12 ‒ 20.65)	13.48 (9.88 ‒ 19.45)
XPNPEP2	88.64 (49.08 ‒ 126.4)#	82.84 (42.9 ‒ 118.58)#	63.26 (32.81 ‒ 113.73)	87.87 (47.1 ‒ 136.02)#	70.07 (46.15 ‒ 103.78)

Controls’ protein levels have previously been published [2,3]. All proteins were measured with proximity extension assay and their levels are expressed in relatively in arbitrary units (AU). Abbreviations: 5′-NT, 5′-nucleotidase; AMBP, protein AMBP (alpha-1-microglobulin/bikunin precursor); AP-N, aminopeptidase N; BLM-H, bleomycin hydrolase; BOC, brother of cell adhesion molecule-related/down-regulated by oncogenes (CDO); CA9, carbonic anhydrase 9; CDKN1A, cyclin-dependant kinase inhibitor 1; CEACAM1 and 5, carcinoembryonic antigen-related cell adhesion molecule 1 and 5; CPA1, B1 and E, carboxypeptidase A1, B1 and E; EpCAM, epithelial cell adhesion molecule; FABP4, fatty acid-binding protein 4; FGF-21 and 23, fibroblast growth factor 21 and 23; FR-gamma, folate receptor gamma; IGF1R, insulin-like growth factor 1 receptor; IGFBP2, 3 and 7, insulin-like growth factor-binding protein 2, 3 and 7; LDL-R, low-density lipoprotein receptor; LOX-1, lectin-like oxidized LDL receptor 1; LPL, lipoprotein lipase; LYPD3, Ly6/PLAUR domain-containing protein 3; MetAP2, methionine aminopeptidase 2; MIA, melanoma-derived growth regulatory protein; PAH, pulmonary arterial hypertension; PCSK9, proprotein convertase subtilisin/kexin type 9; PON-3, paraoxonase-3; (HFpEF/HFrEF)-PH, heart failure with preserved or reduced ejection fraction with pulmonary hypertension; LHF-PH, left heart failure with PH; RARRES2, retinoic acid receptor responder protein 2; S100A11, protein S100A11; S100A4, protein S100A4; SCAMP3, secretory carrier-associated membrane protein 3; SCGB3A2, secretoglobin family 3A member 2; SHPS-1, tyrosine-protein phosphatase non-receptor type substrate 1; sRAGE, soluble receptor for advanced glycation end products; TCL1A, T-cell leukaemia/lymphoma protein 1A; TFF3, trefoil factor 3; TGM2, protein-glutamine gamma-glutamyltransferase 2; TR, transferrin receptor protein 1; WFDC2, WAP four-disulfide core domain protein 2; VSIG2, V-set and immunoglobulin domain-containing protein 2 XPNPEP2, Xaa-Pro aminopeptidase 2.

# n-1; IQR, interquartile range.

**Table 2:** Proteins’ classification and p-values of Kruskal Wallis and Mann Whitney's tests in comparing metabolism and tumour related proteins in controls and disease groups.

	Kruskal Wallis	Control vs HFpEF-PH	Control vs HFrEF-PH	HFpEF-PH vs HFrEF-PH		HFpEF-PH or LHF-PH vs PAH
Protein (AU)	Median (IQR)	Multiple comparisons p-values			Proteins’ Classification	P-value
5′-NT	6.2 × 10^−7^§	0.00042*	9.0 × 10^−9^*	0.045	LHF-PH	0.077
AMBP	0.00026§	6.4 × 10^−5^*	0.056	0.012	HFpEF-PH	0.00022¤
AP-N	0.027	–	–	–	–	–
BLM-H	0.63	–	–	–	–	–
BOC	0.0019§	0.67	0.0026*	0.0034*	–	–
CA9	3.9 × 10^−5^§	2.0 × 10^−5^*	0.00013*	0.53	LHF-PH	0.85
Cathepsin Z	0.025	–	–	–	–	–
CDKN1A	0.024	–	–	–	–	–
CEACAM1	0.00070§	0.11	0.00023*	0.017	–	–
CEACAM5	0.052	–	–	–	–	–
Contactin-1	0.032	–	–	–	–	–
Cornulin	2.1 × 10^−6^§	0.027	6.7 × 10^−7^*	0.0018*	–	–
CPA1	0.0024§	0.071	0.00057*	0.071	–	–
CPB1	0.00025§	0.079	7.2 × 10^−5^*	0.014*	–	–
CPE	0.00030§	0.71	0.00085*	0.00063*	–	–
Cystatin B	7.8 × 10^−7^§	1.01 × 10^−6^*	3.2 × 10^−6^*	0.67	LHF-PH	0.029
Decorin	0.031	–	–	–	–	–
Endocan	2.9 × 10^−7^§	0.00060*	4.0 × 10^−7^*	0.022	LHF-PH	4.3 × 10^−5^¤
EpCAM	0.56	–	–	–	–	–
FABP4	1.3 × 10^−8^§	7.1 × 10^−8^*	4.6 × 10^−8^*	0.93	LHF-PH	0.0035¤
FGF-21	1.4 × 10^−6^§	0.00018	2.7 × 10^−7^*	0.14	LHF-PH	0.21
FGF-23	4.6 × 10^−11^§	2.6 × 10^−6^*	5.9 × 10^−12^*	0.02	LHF-PH	0.028
FR-gamma	0.041	–	–	–	–	–
Furin	0.0022§	0.00072*	0.0045*	0.48	LHF-PH	0.16
Gastrotropin	0.00078§	0.0016*	0.00032*	0.67	LHF-PH	0.00040¤
Glyoxalase I	6.0 × 10^−5^§	4.2 × 10^−5^*	0.00013*	0.66	LHF-PH	2.24 × 10^−7^¤
IGF1R	9.6 × 10^−8^§	3.7 × 10^−5^*	1.7 × 10^−8^*	0.1	LHF-PH	1.7 × 10^−5^¤
IGFBP1	0.00011	0.00040*	4.3 × 10^−5^*	0.61	LHF-PH	0.16
IGFBP2	6.3 × 10^−6^§	4.7 × 10^−6^*	2.5 × 10^−5^*	0.57	LHF-PH	0.44
IGFBP7	6.8 × 10^−10^§	1.8 × 10^−5^*	8.5 × 10^−11^*	0.018	LHF-PH	0.00022¤
Kallikrein 11	2.6 × 10^−7^§	3.7 × 10^−5^*	5.3 × 10^−8^*	0.16	LHF-PH	2.4 × 10^−5^¤
Kallikrein 13	0.0029§	0.0021*	0.0019*	0.96	LHF-PH	0.014
Kallikrein 14	0.056	–	–	–	–	–
Kallikrein 6	0.00010§	0.0085*	1.8 × 10^−5^*	0.072	LHF-PH	0.0035¤
Kallikrein 8	0.12	–	–	–	–	–
LDL-R	0.24	–	–	–	–	–
Leptin	0.0056§	0.0018*	0.18	0.034	–	–
LOX-1	1.0 × 10^−5^§	0.00036*	2.3 × 10^−7^*	0.23	LHF-PH	0.25
LPL	0.0015§	0.0085*	0.79	0.00070*	HFpEF-PH	3.6 × 10^−6^¤
LYPD3	0.0060§	0.0065*	0.0025*	0.78	LHF-PH	0.032
Mesothelin	0.0040§	0.015	0.00098*	0.34	–	–
MetAP2	0.61	–	–	–	–	–
MIA	0.19	–	–	–	–	–
Midkine	1.5 × 10^−5^§	4.3 × 10^−6^*	0.00019*	0.26	LHF-PH	0.27
Mucin 16	0.00020§	0.13	8.7 × 10^−5^*	0.0059*	–	–
Nectin-4	0.00020§	4.5 × 10^−5^*	0.0023*	0.19	LHF-PH	0.16
Pappalysin-1	2.0 × 10^−5^§	0.00013*	8.4 × 10^−6^*	0.55	LHF-PH	0.0014¤
PCSK9	0.013§	0.0082*	0.0073*	0.97	LHF-PH	0.11
Podocalyxin	0.2	–	–	–	–	–
PON3	1.8 × 10^−6^§	1.1 × 10^−5^*	1.4 × 10^−6^*	0.73	LHF-PH	0.73
Prostasin	4.4 × 10^−7^§	6.8 × 10^−7^*	1.8 × 10^−6^*	0.71	LHF-PH	0.25
RARRES2	5.5 × 10^−5^§	1.3 × 10^−5^*	0.00083*	0.19	LHF-PH	0.15
Resisin	0.0010§	0.00021*	0.019	0.095	–	–
S100A11	1.1 × 10^−5^§	6.1 × 10^−6^*	4.8 × 10^−5^*	0.52	LHF-PH	0.91
S100A4	0.035	–	–	–	–	–
SCAMP3	0.066	–	–	–	–	–
SCGB3A2	0.00018§	4.4 × 10^−5^*	0.0017*	0.23	LHF-PH	0.98
SERPIN A12	0.014§	0.17	0.0041*	0.097	–	–
SHPS-1	0.023	–	–	–	–	–
Sortillin	0.00027§	8.9 × 10^−5^*	0.0011*	0.39	LHF-PH	0.056
sRAGE	7.8 × 10^−12^§	0.00075*	2.0 × 10^−12^*	5.0 × 10^−5^*	LHF-pH/HFpEF-PH	0.097#
TCL1A	0.014§	0.023	0.0043*	0.54	–	–
TFF3	2.3 × 10^−8^§	5.9 × 10^−8^*	1.4 × 10^−7^*	0.72	LHF-PH	0.81
TGM2	0.062	–	–	–	–	–
TR	0.018§	0.031	0.0055*	0.52	–	–
WFDC2	3.0 × 10^−9^§	2.8 × 10^−8^*	6.0 × 10^−9^*	0.88	LHF-PH	0.94
Vimentin	8.0 × 10^−8^§	2.4 × 10^−6^*	4.0 × 10^−8^*	0.44	LHF-PH	0.57
VSIG2	7.0 × 10^−9^§	8.0 × 10^−9^*	1.6 × 10^−7^*	0.43	LHF-PH	0.025
XPNPEP2	0.15	–	–	–	–	–

Controls’ protein levels have previously been published [2,3]. Mann Whitney's U tests, Kruskal Wallis tests and following multiple comparison tests were conducted to assess the differences in proteins’ levels between the controls and disease groups. The study comprised 20 controls, 48 pulmonary arterial hypertension (PAH) and 67 left heart failure with pulmonary hypertension (LHF-PH) patients. The latter group included heart failure with preserved/reduced ejection fraction with PH (HFpEF-PH (*n* = 31)/HFrEF-PH (*n* = 36)). Abbreviations: 5′-NT, 5′-nucleotidase; AMBP, protein AMBP (alpha-1-microglobulin/bikunin precursor); AP-N, aminopeptidase N; BLM-H, bleomycin hydrolase; BOC, brother of cell adhesion molecule-related/down-regulated by oncogenes (CDO); CA9, carbonic anhydrase 9; CDKN1A, cyclin-dependant kinase inhibitor 1; CEACAM1 and 5, carcinoembryonic antigen-related cell adhesion molecule 1 and 5; CPA1, B1 and E, carboxypeptidase A1, B1 and E; EpCAM, epithelial cell adhesion molecule; FABP4, fatty acid-binding protein 4; FGF-21 and 23, fibroblast growth factor 21 and 23; FR-gamma, folate receptor gamma; IGF1R, insulin-like growth factor 1 receptor; IGFBP2, 3 and 7, insulin-like growth factor-binding protein 2, 3 and 7; LDL-R, low-density lipoprotein receptor; LOX-1, lectin-like oxidized LDL receptor 1; LPL, lipoprotein lipase; LYPD3, Ly6/PLAUR domain-containing protein 3; MetAP2, methionine aminopeptidase 2; MIA, melanoma-derived growth regulatory protein; PCSK9, proprotein convertase subtilisin/kexin type 9; PON-3, paraoxonase-3; RARRES2, retinoic acid receptor responder protein 2; S100A11, protein S100A11; S100A4, protein S100A4; SCAMP3, secretory carrier-associated membrane protein 3; SCGB3A2, secretoglobin family 3A member 2; SHPS-1, tyrosine-protein phosphatase non-receptor type substrate 1; sRAGE, soluble receptor for advanced glycation end products; TCL1A, T-cell leukaemia/lymphoma protein 1A; TFF3, trefoil factor 3; TGM2, protein-glutamine gamma-glutamyltransferase 2; TR, transferrin receptor protein 1; WFDC2, WAP four-disulfide core domain protein 2; VSIG2, V-set and immunoglobulin domain-containing protein 2 XPNPEP2, Xaa-Pro aminopeptidase 2.

§ *p*<0.020.

⁎ *p*<0.014.

¤ *p*<0.004.

# HFpEF-PH vs PAH AU, arbitrary units IQR, interquartile range False discovery rate (*Q* = 0.01) – p-value is not available due to that the protein not being eligible for further testing sRAGE were classified as HFpEF-PH and LHF-PH protein during the diagnostic and prognostic analyses, respectively.

### 1.3 Tumour and metabolism related proteins in assessing prognosis in HFpEF-PH and/or LHF-PH

To assess the proteins’ crude prognostic performance in identifying events in HFpEF-PH or LHF-PH and to define their optimal plasma thresholds for Kaplan-Meier analyses, ROC analyses were conducted for all 36 proteins comparing death or transplantation (events, *n* = 53) vs event-free survival (non-events, *n* = 14) (Table 3). Endocan had the largest AUC in predicting death or transplantation, followed by sRAGE, IGF1R, FGF-23 and IGFBP7 (Table 3).

**Table 3:** Receiver operating characteristic analysis illustrating the diagnostic and prognostic potential of tumour and metabolism related proteins.

**ROC of proteins differentiating HFpEF-PH from PAH**					
Protein (AU)	total n (events)¤	AUC (95% CI)	Protein	total n (events)¤	AUC (95% CI)
LPL	79 (31)	0.80 (0.70 - 0.90)	Kallikrein 11	79 (31)	0.67 (0.54 - 0.79)
Glyoxalase I	79 (31)	0.78 (0.67 - 0.88)	FABP4	79 (31)	0.65 (0.52 - 0.78)
AMBP	79 (31)	0.74 (0.63 - 0.85)	Endocan	79 (31)	0.64 (0.51 - 0.77)
Gastrotropin	79 (31)	0.68 (0.56 - 0.80)	Pappalysin-1	79 (31)	0.64 (0.52 - 0.77)
IGFBP7	79 (31)	0.68 (0.55 - 0.79)	Kallikrein 6	79 (31)	0.58 (0.45 - 0.72)
IGF1R	79 (31)	0.67 (0.55 - 0.79)			

**ROC of prognostic LHF-PH/HFpEF-PH proteins**					
Protein (AU)	total n (events)#	AUC (95% CI)	Protein (AU)	total n (events)#	AUC (95% CI)

Endocan*	66 (52)	0.77 (0.65 to 0.90)	Glyoxalase I	67 (53)	0.60 (0.46 to 0.74)
sRAGE*	67 (53)	0.75 (0.60 to 0.90)	Vimentin	66 (52)	0.59 (0.42 to 0.77)
IGF1R*	66 (52)	0.73 (0.59 to 0.87)	TFF3	67 (53)	0.59 (0.44 to 0.74)
FGF-23*	67 (53)	0.72 (0.55 to 0.89)	IGFBP2	67 (53)	0.59 (0.44 to 0.74)
IGFBP7*	67 (53)	0.71 (0.55 to 0.87)	FGF-21	67 (53)	0.59 (0.41 to 0.76)
Kallikrein 11	66 (52)	0.70 (0.55 to 0.85)	IGFBP1	67 (53)	0.58 (0.43 to 0.73)
5′-NT	66 (52)	0.67 (0.54 to 0.84)	LPL (HFpEF-PH)	31 (17)	0.58 (0.36 to 0.79)
WFDC2	66 (52)	0.68 (0.54 to 0.82)	LYPD3	66 (52)	0.58 (0.40 to 0.76)
Pappalysin-1	67 (53)	0.66 (0.52 to 0.80)	RARRES2	67 (53)	0.57 (0.39 to 0.75)
PON-3	67 (53)	0.65 (0.47 to 0.83)	SCGB3A2	67 (53)	0.57 (0.41 to 0.73)
Sortillin	67 (53)	0.65 (0.48 to 0.82)	CA9	66 (52)	0.56 (0.39 to 0.73)
Furin	66 (52)	0.65 (0.49 to 0.80)	Kallikrein 13	66 (52)	0.56 (0.41 to 0.71)
PCSK9	67 (53)	0.63 (0.47 to 0.79)	Nectin 4	66 (52)	0.54 (0.38 to 0.71)
FABP4	67 (53)	0.62 (0.44 to 0.80)	Midkine	66 (52)	0.54 (0.39 to 0.68)
Gastrotropin	67 (53)	0.62 (0.46 to 0.79)	AMBP (HFpEF-PH)	31 (17)	0.53 (0.32 to 0.75)
Kallikrein 6	67 (53)	0.61 (0.46 to 0.76)	Prostasin	67 (53)	0.51 (0.34 to 0.69)
Cystatin B	67 (53)	0.61 (0.45 to 0.76)	VSIG2	67 (53)	0.51 (0.33 to 0.69)
LOX-1	67 (53)	0.60 (0.42 to 0.78)	S100A11	66 (52)	0.50 (0.33 to 0.67)

Protein (AU)		Cut-off (AU)		Sensitivity (%)	Specificity (%)

Endocan*		>466.57		75.0%	71.4%
sRAGE*		>44.73		79.2%	71.4%
IGF1R*		>11.76		71.2%	64.3%
FGF-23*		>28.35		90.6%	57.1%
IGFBP7*		>18.73		75.5%	71.4%

The proteins are sorted according to largest area under the roc curve (AUC). In the diagnostic approach, the proteins’ levels that significantly differed heart failure with preserved ejection fraction with pulmonary hypertension (HFpEF-PH) from pulmonary arterial hypertension (PAH) and controls were selected for receiver operating characteristic analysis (ROC). As for the prognostic approach, all 36 proteins that differed left heart failure with PH (LHF-PH) and/or HFpEF-PH from controls were included. The use of HFpEF-PH or the LHF-PH group was according to proteins’ classifications. Abbreviations: 5′-NT, 5′-nucleotidase; AMBP, protein AMBP (alpha-1-microglobulin/bikunin precursor); CA9, carbonic anhydrase 9; CI, confidence interval; FABP4, fatty acid-binding protein 4; FGF-21 and 23, fibroblast growth factor 21 and 23; IGF1R, insulin-like growth factor 1 receptor; IGFBP2, 3 and 7, insulin-like growth factor-binding protein 2, 3 and 7; LOX-1, lectin-like oxidized LDL receptor 1; LPL, lipoprotein lipase; LYPD3, Ly6/PLAUR domain-containing protein 3; PCSK9, proprotein convertase subtilisin/kexin type 9; PON-3, paraoxonase-3; RARRES2, retinoic acid receptor responder protein 2; S100A11, protein S100A11; SCGB3A2, secretoglobin family 3A member 2; sRAGE, soluble receptor for advanced glycation end products; TFF3, trefoil factor 3; WFDC2, WAP four-disulfide core domain protein 2; VSIG2, V-set and immunoglobulin domain-containing protein 2.

¤ PAH (*n* = 48) and HFpEF-PH (31) patients; event=HFpEF-PH diagnosis.

# LHF-PH (*n* = 67) and/or HFpEF patients (*n* = 31); event= death or heart transplantation.

⁎ The five proteins with highest prognostic AUC. Cut-off was defined either with closest top left or Youden's method AU, arbitrary units.

### 1.4 Survival and Cox regression analyses

Patients with LHF-PH with plasma levels >threshold values for endocan, sRAGE, IGF1R, FGF-23 and IGFBP7 had a lower probability of event-free survival compared to levels ≤threshold values (Fig. 1B–F, log-rank *p* < 0.05). In the univariable Cox regression models, IGF1R was the strongest predictor of events per unit increase, followed by sRAGE, IGF1R, endocan and FGF-23 (*p* < 0.05). However, after adjustment for age, sex, atrial fibrillation, and systemic hypertension in multivariable models, the prespecified biomarkers were no longer significantly associated with events (*p* > 0.05), although plasma sRAGE (HR 1.017, 95% CI 0.99–1.036; *p* = 0.07) displayed such propensity (Table 4).

**Table 4:** Univariable and multivariable Cox proportional hazards regression models of transplantation-free survival in left heart failure patients with pulmonary hypertension.

**Univariable Cox regression**									
Predictors	n (events)	HR	95% CI	p-value	Predictors	n (events)	HR	95% CI	p-value
Age (years)	67 (53)	0.97	0.96 - 0.99	0.0011*	Endocan (AU)	66 (52)	1.001	1.00 - 1.003	0.010*
Sex (female)	67 (53)	0.47	0.26 - 0.84	0.011*	FGF-23 (AU)	67 (53)	1.001	1.00 - 1.001	0.012*
Atrial fibrillation (yes)	67 (53)	0.52	0.30 - 0.91	0.021*	IGF1R (AU)	66 (52)	1.057	1.016 - 1.10	0.0063*
Diabetes mellitus (yes)	64 (52)	0.80	0.41 - 1.56	0.50	IGFBP-7 (AU)	67 (53)	1.011	1.001 - 1.021	0.031*
Systemic hypertension (yes)	63 (51)	0.37	0.20 - 0.68	0.0013*	sRAGE (AU)	67 (53)	1.030	1.01 - 1.043	0.0015*

**Multivariable Cox regression**									
Predictors	n (events)	HR	95% CI	p-value	Predictors	n (events)	HR	95% CI	p-value

Endocan (AU)	61 (50)	1.00070	0.99 - 1.0020	0.32	IGFBP-7 (AU)	62 (51)	1.0038	0.99 - 1.017	0.55
Age (years)		0.99	0.96 - 1.0070	0.19	Age (years)		0.98	0.96 - 1.0051	0.14
Sex (female)		0.82	0.40 - 1.69	0.59	Sex (female)		0.87	0.42 - 1.80	0.71
Atrial fibrillation (yes)		0.73	0.34 - 1.55	0.41	Atrial fibrillation (yes)		0.73	0.35 - 1.52	0.40
Systemic hypertension (yes)		0.55	0.27 - 1.11	0.097	Systemic hypertension (yes))		0.50	0.25 - 0.98	0.042*
FGF-23 (AU)	62 (51)	1.00	0.99 - 1.00070	0.94	sRAGE (AU)	62 (51)	1.017	0.99 - 1.036	0.070
Age (years)		0.98	0.96 - 1.0053	0.14	Age (years)		0.98	0.96 - 1.0050	0.14
Sex (female)		0.82	0.41 - 1.66	0.58	Sex (female)		0.86	0.43 - 1.70	0.67
Atrial fibrillation (yes)		0.76	0.37 - 1.58	0.46	Atrial fibrillation (yes)		0.86	0.41 - 1.80	0.68
Systemic hypertension (yes)		0.49	0.25 - 0.95	0.036*	Systemic hypertension (yes)		0.57	0.28 - 1.15	0.12
IGF1R (AU)	61 (50)	1.032	0.023 - 1.39	0.17	Endocan (AU)	66 (52)	1.00	0.99 - 1.0020	0.97
Age (years)		0.98	0.99 - 1.079	0.10090	FGF-23 (AU)		1.00050	0.99 - 1.0010	0.26
Sex (female)		0.77	0.96 - 1.0030	0.46	IGF1R (AU)		0.99	0.93 - 1.055	0.74
Atrial fibrillation (yes)		0.84	0.38 - 1.55	0.64	IGFBP-7 (AU)		1.0057	0.98 - 1.028	0.60
Systemic hypertension (yes)		0.52	0.26 - 1.024	0.060	sRAGE (AU)		1.030	0.99 - 1.056	0.051

Univariable and multivariable Cox regression analyses were conducted for the five proteins that rendered the largest crude area under the roc curve in differentiating event occurrence from event-free survival in left heart failure with pulmonary hypertension (*n* = 67) or heart failure with preserved ejection fraction with pulmonary hypertension (*n* = 31) patients, according to proteins’ classifications. Five predictors were included in each multivariable model due to the limited number of events (*n* = 53) and adjustments were done for age, sex and the two most common comorbidities i.e., atrial fibrillation and systemic hypertension. Abbreviations: AU, arbitrary units; CI, confidence interval; FGF-23, fibroblast growth factor 23; HR, hazard ratio; IGF1R, insulin-like growth factor 1 receptor; IGFBP-7, insulin-like growth factor-binding protein 7; sRAGE, soluble receptor for advanced glycation end products.

⁎ *p* < 0.05 Event= death or heart transplantation.

### 1.5 Other potential prognostic proteins in HFpEF-PH or LHF-PH

Other significant and potentially prognostic markers for transplantation or death in HFpEF-PH or LHF-PH (AUC≤0.70) were kallikrein 11, 5′-NT, WFDC2 and pappalysin-1 (*p* < 0.05; Table 3).

### 1.6 Endocan, sRAGE, IGF1R, FGF-23 and IGFBP7 correlate with NT-proBNP and haemodynamics

In LHF-PH patients, Endocan and FGF-23 correlated with the largest number haemodynamic parameters, followed by IGFBP7, sRAGE and IGF1R (FDR=0.05; Table 5).

**Table 5:** Spearman's correlation analyses of potential prognostic or diagnostic biomarker candidates with NT-proBNP and hemodynamic parameters.

	NT-proBNP (AU)		mPAP (mmHg)		PAWP (mmHg)		MRAP (mmHg)		CI (L/min/m^2^)		PVR (WU)		PAC (mL/mmHg)		RVSWI (g/beat/m^2^)		LVSWI (g/beat/m^2^)	
Variable	n	r (p-value)	n	r (p-value)	n	r (p-value)	n	r (p-value)	n	r (p-value)	n	r (p-value)	n	r (p-value)	n	r (p-value)	n	r (p-value)
AMBP (AU)	31	−0.26(0.17)	31	−0.38(0.036)	31	−0.42(0.018*)	31	−0.31(0.087)	31	0.24(0.19)	31	−0.33(0.070)	31	0.35(0.050)	31	0.025(0.89)	31	0.34(0.058)
LPL (AU)	31	−0.2(0.27)	31	−0.23(0.22)	31	−0.11(0.55)	31	−0.31(0.092)	31	−0.095(0.61)	31	−0.076(0.69)	31	0.053(0.78)	31	−0.066(0.73)	31	0.093(0.62)
Endocan (AU)	66	0.55(1.4 × 10^−6^*)	66	0.28(0.021*)	65	0.22(0.076)	66	0.41(0.00065*)	66	−0.29(0.020*)	65	0.29(0.020*)	66	−0.26(0.038)	66	−0.2(0.11)	65	−0.41(0.00074*)
FGF-23 (AU)	67	0.71(1.5 × 10^−11^*)	67	0.23(0.056)	66	0.29(0.017*)	67	0.68(2.0 × 10^−10^*)	67	−0.3(0.012*)	66	0.068(0.59)	67	0.0079(0.95)	67	−0.38(0.0014*)	66	−0.43(0.00036*)
IGFBP-7 (AU)	67	0.67(4.0 × 10^−10^*)	67	0.34(0.045*)	66	0.29(0.018*)	67	0.64(3.9 × 10^−9^*)	67	−0.23(0.063)	66	0.27(0.028)	67	−0.094(0.45)	67	−0.24(0.054)	66	−0.37(0.0023*)
sRAGE (AU)	67	0.56(6.9 × 10^−7^*)	67	0.18(0.15)	66	0.43(0.00035*)	67	0.17(0.17)	67	−0.54(2.4 × 10^−6^*)	66	0.059(0.64)	67	−0.15(0.23)	67	−0.33(0.0065*)	66	−0.51(1.3 × 10^−5^*)
Glyoxalase I (AU)	31	−0.13(0.48)	31	0.023(0.9)	31	0.26(0.16)	31	−0.28(0.13)	31	0.27(0.15)	31	−0.34(0.059)	31	0.32(0.081)	31	0.27(0.14)	31	0.25(0.18)
IGF1R (AU)	66	0.5(2.0 × 10^−5^*)	66	0.31(0.010*)	65	0.14(0.25)	66	0.39(0.0011*)	66	−0.13(0.3)	65	0.26(0.039)	66	−0.081(0.52)	66	−0.12(0.34)	65	−0.24(0.058)

Spearman's correlation analyses were conducted between HFpEF-PH or the LHF-PH group with NT-proBNP and hemodynamic parameters. The patient groups included in the analysis were determined by proteins’ classifications. False discovery rate was done to accommodate for mass-significance. Abbreviations: AMBP, Protein AMBP (alpha-1-microglobulin/bikunin precursor); AU, arbitrary units; CI, cardiac index; FGF-23, fibroblast growth factor 23; HFpEF-PH; heart failure with preserved ejection fraction with pulmonary hypertension; IGF1R, insulin-like growth factor 1 receptor; IGFBP-7, insulin-like growth factor-binding protein 7; LHF-PH, left heart failure with PH; LPL, lipoprotein lipase; LVSWI, left ventricular stroke work index; mPAP, mean pulmonary arterial pressure; MRAP, mean right atrial pressure; NT-proBNP, N-terminal pro b-type natriuretic peptide; PAC, pulmonary arterial compliance; PAWP, pulmonary arterial wedge pressure; PVR, pulmonary vascular resistance; RVSWI, right ventricular stroke work index; sRAGE, soluble receptor for advanced glycation end products; WU, wood units.

⁎ *p* < 0.024; false discovery rate (*Q* = 0.05) *r* = Spearman's rank correlation coefficient.

## 2 Experimental Design, Materials and Methods

### 2.1 Study population

The study was based on Lund Cardio Pulmonary Registry (LCPR) a unique, single centre prospective cohort in Region Skåne's biobank, initiated by Göran Rådegran in 2011. LCPR contains blood samples of healthy controls, as well as haemodynamic- and clinical data of patients evaluated for pulmonary hypertension (PH) and heart transplantation in a PH-expert centre. The haemodynamic and plasma samples were collected between 2011 and 2017 at Skåne University hospital during the routine clinical assessments. The blood samples were stored at Region Skåne's biobank, Lund, Sweden. Transplantation free survival of patients with LHF-PH were followed-up until 2020.

In the present study, a total of 135 participants were included, comprising healthy controls (*n* = 20) devoid from cardiovascular disease, as well as patients with pulmonary arterial hypertension (PAH) and LHF-PH (*n* = 67); [HFpEF-PH, (*n* = 31) and HFrEF-PH (*n* = 36)]) [1]. A detailed description of the study population has been described elsewhere [1]. The plasma samples were analysed with proximity extension assay, a multiplex immunoassay based on the use of protein-specific pairs of oligonucleotide-linked antibodies and microfluidic qPCR for detection and quantification, conferring high specificity and sensitivity. The proteins were analysed using the Proseek Multiplex Cardiovascular II, III and Oncology II 96-plex immunoassays (Olink, Proteomics, Uppsala, Sweden). Per default, the proteins’ levels are expressed in arbitrary log2 NPX scale, which is a relative quantification scale corresponding to the inverted Ct-value [1]. Due to non-normally distributed log2 scale values and for interpretation purposes, the conventional log2 values were linearized using the following formula: linear NPX (AU) = 2^(log2 NPX)^. The proteins were analysed using the Proseek Multiplex Cardiovascular II, III and Oncology II 96-plex immunoassays (Olink, Proteomics, Uppsala, Sweden) [4].

### 2.2 Validation of the analytical performance

The multiplex analyses of the 96-pelx immunoassays (Cardiovascular II, III and Oncology II) were performed, each by using a separate plate. For quality control, internal and external controls were used. In each well of the three plates, four internal controls were added for quality control of immunoreaction, extension, amplification, and detection. Additionally, external controls, comprising inter-plate controls and negative controls were added, each in three separate wells in every plate. The median of the inter-plate controls was used to monitor and normalize potential variation between runs and plates, whereas the negative controls, consisting of buffer with no antigens, were used to monitor potential background noise and to establish the proteins background levels (calculate the limit of detection). Validation of the analytical performance has been conducted for each panel's measuring range, specificity, sensitivity, precision, reproducibility and scalability (Olink proteomics, Uppsala, Sweden) [4].

Although detection range and standardized curves “calibration curves” are available for almost all proteins – established during the validation of the analytical performance – these can only estimate the expected measuring range of the immunoassays and cannot be used to convert arbitrary NPX to absolute concentrations. Thus, detection range and standardized curves or (calibration curves), specifically for the current analyses, are not available. Nevertheless, standardized curves are not a requisite for interpretation as the proteins are measured in a relative quantification scale. Panel and protein specific validation data for Cardiovascular II, III and Oncology II panels can be downloaded at www.olink.com/downloads(Olink proteomics, Uppsala, Sweden) [4].

### 2.3 NT-proBNP, tumour and metabolism related proteins

NT-proBNP and 69 tumour and metabolism related proteins were analysed: 5′-nucleotidase (5′-NT), protein AMBP or alpha-1-microglobulin/bikunin precursor (AMBP), aminopeptidase N (AP-N), bleomycin hydrolase (BLM-H), brother of cell adhesion molecule-related/down-regulated by oncogenes (CDO) or (BOC), carbonic anhydrase 9 (CA9), cathepsin Z, cyclin-dependant kinase inhibitor 1A (CDKN1A) or p21, carcinoembryonic antigen-related cell adhesion molecule (CEACAM) 1 and 5, contactin-1, cornulin, carboxypeptidase (CP) A1, B1 and E, cystatin B, decorin, endothelial cell-specific molecule 1 or endocan, epithelial cell adhesion molecule (EpCAM), fatty acid-binding protein 4 (FABP4), fibroblast growth factor (FGF-) 21 and 23, folate receptor gamma (FR-gamma), furin, gastrotropin, glyoxalase I or lactoylglutathione lyase, insulin-like growth factor 1 receptor (IGF1R), insulin-like growth factor-binding protein (IGFBP) 2, 3 and 7, kallikrein 6, 8, 11, 13 and 14, low-density lipoprotein receptor (LDL-R), leptin, lectin-like oxidized LDL receptor 1 (LOX-1), lipoprotein lipase (LPL), Ly6/PLAUR domain-containing protein 3 (LYPD3) or C4.4A, mesothelin, methionine aminopeptidase 2 (MetAP2), melanoma-derived growth regulatory protein (MIA), midkine, mucin-16 or CA125, nectin-4 or PVRL4, pappalysin-1, proprotein convertase subtilisin/kexin type 9 (PCSK9), podocalyxin, paraoxonase-3 (PON-3), prostasin, protein S100A4 (S100A4), protein S100A11 (S100A11), retinoic acid receptor responder protein 2 (RARRES2), resistin, secretory carrier-associated membrane protein 3 (SCAMP3), secretoglobin family 3A member 2 (SCGB3A2), serpin A12, tyrosine-protein phosphatase non-receptor type substrate 1 (SHPS-1), sortillin, soluble receptor for advanced glycation end products (sRAGE; all soluble forms of RAGE), T-cell leukaemia/lymphoma protein 1A (TCL1A), trefoil factor 3 (TFF3), protein-glutamine gamma-glutamyltransferase 2 (TGM2), transferrin receptor protein 1 (TR), WAP four-disulfide core domain protein 2 (WFDC2), vimentin, V-set and immunoglobulin domain-containing protein 2 (VSIG2) and Xaa-Pro aminopeptidase 2 (XPNPEP2).

### 2.4 Clinical evaluation and haemodynamic definitions

The clinical evaluations, diagnosis were made by experienced cardiologists in a national PH and heart transplantation referral centre, at the “Haemodynamic lab” at Skåne University hospital in Lund, Sweden, in accordance with contemporary guidelines [5,6]. Between October 2011 and February 2016, patients’ haemodynamics were measured with right heart catheterisation in supine position through inserting a Swan Ganz catheter (Baxter Health Care Corp, Santa Ana, CA) via an introducer preferentially in the right internal jugular vein. Vasoreactivity testing was performed with inhaled nitric oxide in PAH de novo patients, and with intravenous nitroprusside infusion in HF patients with elevated pulmonary vascular resistance (PVR) during evaluation for heart transplantation. Cardiac output (CO) was estimated with thermodilution and heart rate was measured with electrocardiography. Mean arterial pressure was assessed non-invasively and calculated as 1/3 systolic + 2/3 diastolic blood pressure.

Body surface area, CO, systolic, diastolic and mean pulmonary arterial pressures (sPAP, dPAP and mPAP), PAWP and mean right atrial pressure (MRAP) were used to calculate additional parameters using the following formulas: cardiac index= CO/body surface area, stroke volume (SV)= CO/heart rate, SV index (SVI)= SV/body surface area, left ventricular stroke work index= (mean arterial pressure-PAWP) × SVI, right ventricular stroke work index= (mPAP-MRAP) × SVI, PVR= (mPAP-PAWP)/CO, PVR index=(mPAP-PAWP)/cardiac index, pulmonary arterial compliance= SV/(sPAP-dPAP).

PAH was defined in relation to the present guidelines at the time as precapillary PH (mPAP ≥ 25 mmhg, PAWP≤ 15 mmhg and a PVR>3 wood units (WU)), after excluding other aetiologies of pre-capillary PH through using a multimodal diagnostic approach, including computed and high-resolution tomography, echocardiography and/or magnetic resonance imaging, pulmonary scintigraphy and spirometry with diffusion capacity. PH due to left heart failure was defined with a resting mPAP ≥ 25 mmhg and a PAWP > 15 mmhg, subclassified into isolated post-capillary PH (DPG < 7 mmHg and/or PVR ≤3 WU) or combined post-capillary and pre-capillary PH (DPG ≥7 and/or PVR >3 WU) [6]. Left ventricular systolic and diastolic dysfunction were diagnosed with echocardiography and/or magnetic resonance imaging. The World Health Organisation functional class, 6 min walking distance, and other clinical parameters including mixed venous oxygen saturation, arterial oxygen blood saturation and arteriovenous oxygen difference were measured or performed in conjunction with the haemodynamic assessments. Patients’ demographics were retrieved from the LCPR file. The estimated glomerular filtration rate was creatinine-based and calculated with the revised Lund-Malmö formula [7].

### 2.5 Statistical analyses

Statistical tests were performed with R version 4.0.2, (Foundation for Statistical Computing, Vienna, Austria) and GraphPad Prism version 8.4.1 for Windows, (GraphPad Software, San Diego, California USA. Normality was assessed visually with histograms, and data is presented median (inter quartile range), unless stated otherwise. Due to the dominance of non-normally distributed data, non-parametric tests were employed. Kruskal Wallis tests with a following multiple comparison post-hoc analyses in addition to Mann-Whitney U tests were used to assess the differences in protein's levels between groups. To correct for mass significance, and to define significance threshold, false discovery rate (FDR) was used. Statistically significant results were either defined as p-values less than the achieved thresholds, or when FDR were not applicable, as *p* < 0.05. Area under the ROC curve (AUC) was used to choose the best prognostic proteins (highest AUC). Youden's index or closest distance to top left were used to define the ideal thresholds for potential prognostic proteins. Kaplan-Meier method was used to estimate event-free survival in relation to potential prognostic biomarkers during the overall follow-up time, and the log-rank test to compare transplantation-free survival between groups. Transplantation-free survival was defined as survival free from death or transplantation. Censoring was applied to the end-of-study follow-up time. Assumptions of proportional hazards were graphically examined, and Cox proportional hazards regressions were used to estimate the univariable and the multivariable hazard ratios (HR) of potential prognostic biomarkers. Due to the limited number of events, five independent variables were used to adjust for age, sex and most common comorbidities in the Cox regression analyses. Spearman's rank correlation was used to associate NT-proBNP and haemodynamics with the plasma levels of potential prognostic biomarkers.

### 2.6 Eligible proteins for prognostic analyses

Briefly, the criteria applied to identify proteins eligible for diagnostic analyses were also applied to identify proteins eligible for prognostic analyses. The criteria are described more in detail elsewhere [1].

## Ethics Statements

All participants signed a written informed consent upon enrolment. The study has been performed in accordance with the declaration of Helsinki and Istanbul and was approved by the local ethical board in Lund, Sweden (Dnr: 2010/114, 2010/442, 2011/368, 2011/777, 2014/92 and 2015/270).

## CRediT authorship contribution statement

**Salaheldin Ahmed:** Conceptualization, Methodology, Software, Validation, Investigation, Writing – original draft, Formal analysis, Visualization. **Abdulla Ahmed:** Conceptualization, Methodology, Visualization, Investigation, Writing – review & editing. **Göran Rådegran:** Conceptualization, Methodology, Investigation, Resources, Writing – review & editing, Supervision, Project administration, Funding acquisition.

## Declaration of Competing Interest

The authors declare that they have no known competing financial interests or personal relationships that could have appeared to influence the work reported in this paper.

The authors declare the following financial interests/personal relationships which may be considered as potential competing interests:

Dr. Salaheldin Ahmed and Dr. Abdulla Ahmed report personal lecture fees from Janssen-Cilag AB. Dr. Rådegran reports personal lecture fees from Actelion Pharmaceuticals Sweden AB, Bayer Health Care, GlaxoSmithKline, Janssen-Cilag AB and Nordic Infucare outside the submitted work.

Dr. Rådegran reports unrestricted research grants from ALF, a non-interventional investigator-initiated study research grant from Janssen-Cilag AB during the conduct of the study.

Dr. Rådegran is, and has been primary-, or co-, investigator in; clinical PAH trials for Actelion Pharmaceuticals Sweden AB, Bayer, GlaxoSmithKline, Pfizer, Janssen-Cilag AB and United Therapeutics, and in clinical heart trans- plantation immuno-suppression trials for Novartis.

The relationship with the organisations described above played no role in the collection, analysis or interpretation of the data, had no right to restrict the publishing of the manuscript, and did not impose a competing interest.
